# Variable meal stimulation may mimic pancreatic dysregulation in malnourished children

**DOI:** 10.1113/EP094416

**Published:** 2026-10-22

**Authors:** Erdem Topal, Ercan Yılmaz, Nezihe Koker Özer

**Affiliations:** ^1^ Pediatric Allergy and Immunology Department, Faculty of Medicine Inonu University Malatya Turkey

**Keywords:** Children, dysregulation, pancreas

1

To the Editor,

We read with interest the study by Besa et al. (2026) examining pancreatic and gastrointestinal hormone responses to feeding in Zambian children with severe acute malnutrition (SAM). The authors report a higher proportion of ‘disordered’ insulin and C‐peptide responses in SAM and interpret these findings as evidence of impaired pancreatic regulation that could reduce the effectiveness of standard feeding therapy. We believe that an unaddressed exposure–outcome problem might substantially alter this interpretation, that is, the hormonal response was classified after a nutrient stimulus that was neither standardized to body size nor incorporated into the analysis.

The three groups did not receive an equivalent physiological stimulus. Hospitalized control subjects consumed 150 mL of PediaSure, whereas SAM and community children received F100 ad libitum, with median intakes of 120 and 175 mL, respectively. Thus, the amount of nutrient delivered (and potentially carbohydrate, protein and energy exposure per kilogram) could vary systematically with disease status and appetite. In children with SAM, reduced appetite is itself part of the clinical phenotype; consequently, a smaller ingested stimulus might generate a smaller insulin/C‐peptide increment even when β‐cell responsiveness is preserved. Standardized mixed‐meal testing uses a controlled nutrient stimulus to make stimulated C‐peptide responses interpretable across individuals (Greenbaum et al., 2008).

This concern is amplified by the definition of a ‘disordered’ response. Any insulin or C‐peptide delta ≤ 0 was classified as abnormal, although the reported interassay coefficients of variation were 8.04% for insulin and 2.03% for C‐peptide. A trivial negative change within analytical and within‐person variability can therefore cross the abnormality threshold. Reference‐change methodology emphasizes that serial biomarker differences should exceed expected analytical and biological variation before being interpreted as physiologically meaningful (Fraser, 2011). Moreover, SAM children began with substantially higher basal insulin and C‐peptide concentrations than comparators. When pre‐existing groups differ at baseline, change‐score and baseline‐adjusted analyses can yield materially different conclusions (Van Breukelen, 2006).

Clinically, these issues matter because reduced nutrient intake combined with a zero‐threshold delta might transform an inadequate stimulus into apparent pancreatic dysfunction. Before inferring intrinsic β‐cell impairment (or considering targeted metabolic interventions) we suggest sensitivity analyses incorporating the real nutrient exposure (preferably as kilocalories per kilogram) and macronutrient load, alongside continuous postprandial insulin and C‐peptide outcomes accounting for baseline values. Reclassifying abnormal responses using a threshold exceeding analytical and biological variability would provide an additional robustness test. If the excess ‘disordered’ responses in SAM persist after these analyses, the evidence for intrinsic pancreatic dysregulation would be considerably stronger.

## AUTHOR CONTRIBUTIONS

Erdem Topal: Supervision, conceptualization. Ercan Yılmaz: Supervision, conceptualization; writing—original draft. Nezihe Koker Ozer: Supervision; conceptualization; writing—original draft. All authors approved the final version of the manuscript and agree to be accountable for all aspects of the work in ensuring that questions related to the accuracy or integrity of any part of the work are appropriately investigated and resolved. All persons designated as authors qualify for authorship, and all those who qualify for authorship are listed.

## CONFLICT OF INTEREST

None declared.

## FUNDING INFORMATION

None.

## GENERATIVE AI STATEMENT

The authors used Generative AI (ChatGPT, OpenAI) solely for English language editing and grammatical improvement. It was not used to generate scientific content, analyse data, interpret results or draw conclusions.
